# Editorial: Utilization of microbiome to develop disease resistance in crop plants against phytopathogens

**DOI:** 10.3389/fpls.2023.1204896

**Published:** 2023-05-26

**Authors:** Srayan Ghosh, Gopaljee Jha

**Affiliations:** ^1^Department of Biosciences, Durham University, Durham, United Kingdom; ^2^Plant-microbe interactions lab, National Institute of Plant Genome Research, Delhi, India

**Keywords:** microbiome, phytopathogen, biocontrol, antagonists, disease suppresion

Sustainable agriculture is important for ensuring food security at an affordable cost. Agricultural practices heavily depend on agrochemicals, such as fertilizers, fungicides, and pesticides, to ensure optimal productivity and prevent losses due to pathogens and pests. However, with time, it has become clear that most commonly used agrochemicals have residual side effects on consumers, soil health, and the environment. Hence, developing newer environmentally friendly approaches to ensure crop productivity, with minimal usage of agrochemicals, is highly desirable. The potential of plant/rhizospheric soil-associated microbes to support the overall growth of plants and provide protection against phytopathogens has been actively studied in recent years (Busby et al., 2017; Liu et al., 2020). A large number of beneficial bacteria, including Bacillus, Pseudomonads and Burkholderia sp. and fungi, including Trichoderma sp. and *Piriformospora indica*, have been shown to have growth-promoting and disease-protection abilities (Hacquard et al., 2017). It is to be noted that billions of microbes live within or in very close association with plants; together, they influence the growth and immunity of the host plant. Moreover, due to intraspecific or intraspecific competitions, there is a constant flux in the population dynamics of the microbiota (Das et al., 2021). Hence it is important to understand the interactions among the plant-associated microbes to comprehend their ecological fitness under field conditions. The genome sequence of plant-associated beneficial microorganisms has unravelled that they are enriched in novel antimicrobial compounds and secondary metabolite-encoding biosynthetic gene clusters (BGCs), however, most of them remain to be characterised. Characterising the coding potential of plant-associated microbes will profusely contribute to the global bio-economy.

This Research Topic brings four important articles that exemplify and emphasise the utility of plant-associated microbes in sustainable agriculture (**Figure 1**). Lee et al. report that a gram-positive bacterium, *Bacillus velezensis* GH1-13, helps manage brown patch disease of cool-season turfgrass caused by a necrotrophic fungal pathogen, *Rhizoctonia solani*. The study demonstrates that the application of GH1-13 helps reduce the usage of azoxystrobin fungicide to 50%, as GH1-13 + 50% of azoxystrobin provided a similar level of protection to that observed in the case of 100% of azoxystrobin treatment, even under field conditions. In addition, they have developed a qRT-PCR-based assay to precisely detect the GH1-13. The analysis reflected that the bacterium could stably cohabit and survive in the rhizosphere suggesting its use as a stable biocontrol agent. This highlights that the focus should be on developing more straightforward approaches to quantify the ecological fitness of the microbes under field conditions.

**Figure 1:**
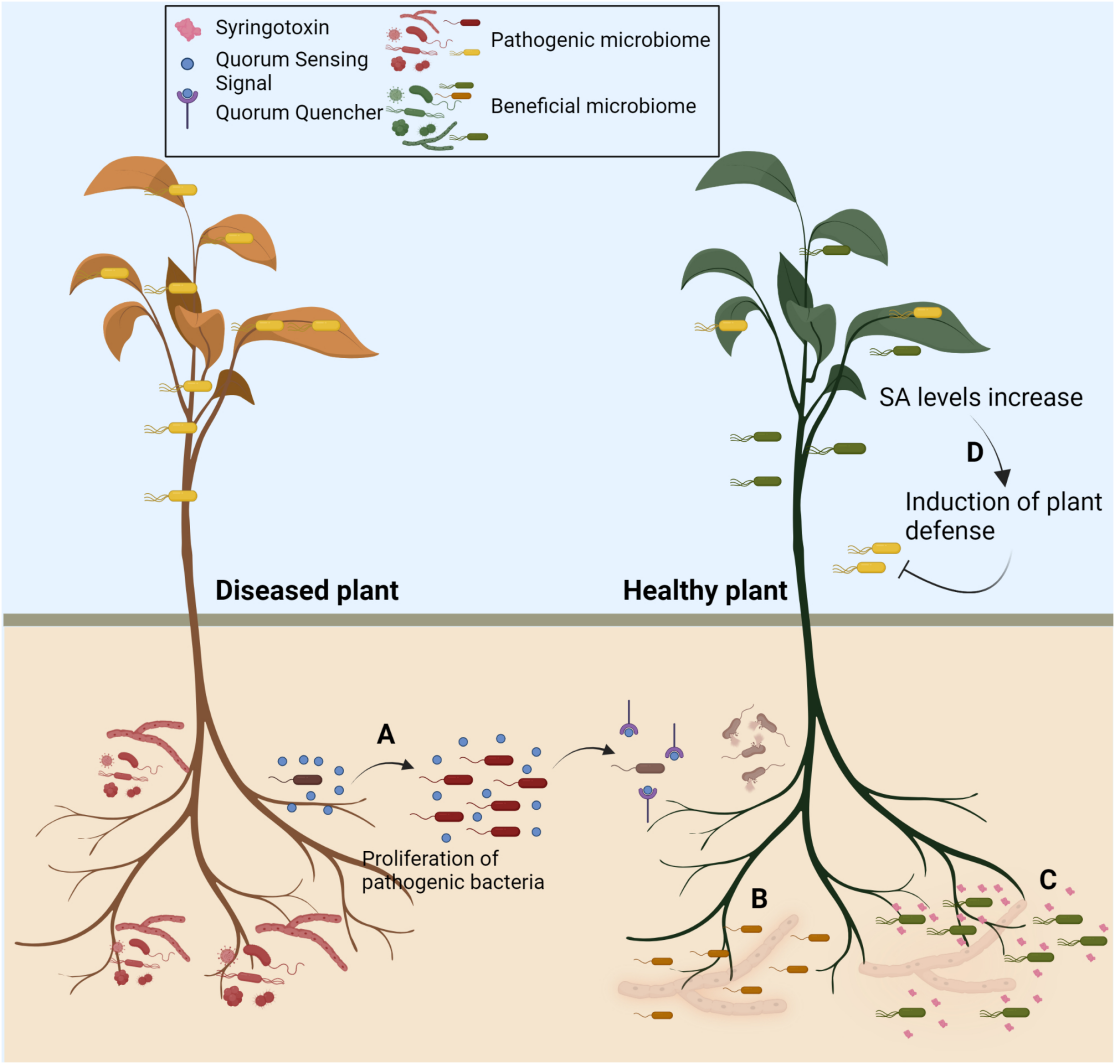
Strategies to deploy microbiome for crop improvement. Recent studies have highlighted that plant-associated microbes and the microbiome can be harnessed for crop protection. The articles in the research topic exemplify **(A)** Use of quorum quenching to disrupt the quorum sensing of the pathogenic bacteria to prevent disease **(B)** Use of antagonistic bacteria to protect plants from fungal diseases **(C)** Some novel secondary metabolites secreted from the plant-associated bacteria can antagonise fungal pathogens **(D)** Microbiome-mediated priming of Salicylic acid-mediated plant defence to prevent subsequent attacks from pathogenic microorganisms. Created with BioRender.com.

Ferrarini et al. have characterised the role of the biosynthetic gene clusters (BGCs) associated with the synthesis of cyclic lipopeptides (CLPs) in a phytopathogenic bacterium *Pseudomonas fuscovaginae* UPB264 that causes sheath brown rot disease in rice. The authors have obtained a single-contig genome of the UPB264 and identified that the bacterium encodes novel CLPs, asplenin, in addition to the previously reported CLPs, syringotoxin and fuscopeptin. Using the mutant strains, fuscopeptin and syringotoxin are important for the bacterium causing rice sheath rot disease. In contrast, asplenin and syringotoxin are important for bacterial swarming motility and antifungal activity. Syringotoxin has been reported to have antifungal activity against sheath blight pathogen *Rhizoctonia solani.* The study emphasises the characterisation of putative BGCs is an important area of research, which would lead to the identification of novel bioactive molecules for potential exploitation for plant growth promotion and disease control.

One of the important attributes of beneficial microbes is their ability to induce host immune responses and prevent disease establishment by phytopathogens. It is important to understand the defence-priming ability of the beneficial microbes to utilise them for disease control. Li et al. report that a *Priestia megaterium* strain JR48, isolated from the vegetable field, has a plant-growth-promoting and biocontrol effect. The data reflected that bacterial Treatment not only enhances the Chinese cabbage’s growth but also prevents the plants from black rot disease caused by *Xanthomonas campestris* pv. campestris 8004 (Xcc) infection. Notably, induced expression of various defence-related genes, including *pathogenesis-related* (*PR*) genes, enhanced callose deposition, salicylic acid (SA) accumulation, and hydrogen peroxide, were observed in the JR48-treated plants. Overall, the study emphasizes the importance of salicylic acid (SA)- mediated defence in the biocontrol ability of JR48.

Notably, plant-associated microbes continuously interact with the host plants and other co-having microbes in a hostile environment. They have intricate ability to communicate with each other using quorum sensing molecules. Extensive progress has been made in understanding the quorum-sensing mechanism and exploring whether using quorum-quenching molecules could prevent phytopathogens from establishing disease. Zhu et al. provide the current understanding in this field and elaborate on how quorum quenching is effective in countering pathogen growth and attenuating virulence in crop plants. The quorum quenchers either function through competitive inhibition of the active site or physical degradation of the signalling molecules. They are primarily considered to have fewer side effects on non-target micro-organisms. An additional advantage of using quorum quenchers is that, unlike antibiotics, for which there is selection pressure in bacteria to develop resistance, it is challenging to build tolerance against quorum quenchers. The review not only elaborates on various applications of quorum quenchers in agriculture but also discusses their exploitation in controlling aquacultural pathogens, human pathogens, and biofouling mitigation. The article advocates that it is important to investigate quorum quenching molecules’ side effects on non-target organisms, particularly whether they can alter the colonisation of beneficial microorganisms. The special issue on utilising microbiome for controlling phytopathogens provide novel approaches for biocontrol of diseases.

## Author contributions

All authors listed have made a substantial, direct, and intellectual contribution to the work and approved it for publication.
